# Screening of Different Extracts from *Artemisia* Species for Their Potential Antimalarial Activity

**Published:** 2015

**Authors:** Mahdi Mojarrab, Rozhin Naderi, Fariba Heshmati Afshar

**Affiliations:** a *Novel Drug Delivery Research Center, School of Pharmacy, Kermanshah University of Medical Sciences, Kermanshah, Iran.*; b *Student Research Committee, Kermanshah University of Medical Sciences, Kermanshah,Iran. *; c *Drug Applied Research Center, Tabriz University of Medical Sciences, Tabriz, Iran.*

**Keywords:** *β*-*hematinformation*, *Artemisiaciniformis*, *Artemisiabiennis*, *Artemisiaturanica*, Antimalaria

## Abstract

The formation of hemozoin (malaria pigment) has been proposed as an ideal drug target for antimalarial screening programs. In this study, we used an improved, cost-effective and high-throughput spectrophotometric assay to screen plant extracts for finding novel antimalarial plant sources. Fifteen extracts with different polarity from three Iranian *Artemisia* species, *A. ciniformis*, *A. biennis* and *A. turanica,* were assessed for their antimalarial activity by *in-vitro β*-hematin formation assay. The most potent effect was observed in dichloromethane (DCM) extract of *A. ciniformis *with IC_50_ and IC_90_ values of 0.92 ± 0.01 and 1.29 ± 0.02 mg/mL, respectively**.** Ethyl acetate (EtOAC) extracts of *A. biennis* and *A. turanica* also showed significant antimalarial activities with IC_50_ values of 1.11 ± 0.02 and 1.35 ± 0.08 mg/mL and IC_90_ values of 1.22 ± 0.04 and 2.81 ± 0.21 mg/mL, respectively. Based on these results, it is possible to conclude that the components with strong antimalarial activity have been concentrated in the medium-polar extracts.

## Introduction

Malaria continues to be a life threatening disease in the tropical and subtropical regions with the strongest mortality (1). It is transmitted by protozoa of the genus *plasmodium *and responsible for hundreds of millions of infections that kill between one and three million people annually (2). This situation has been complicated by the emergence of parasite strains resistant to the existing inexpensive drugs such as chloroquine (3); therefore, there is an urgent need to find alternative drugs especially traditional and herbal remedies for the treatment of the disease. Members of the genus *Artemisia* (Asteraceae) are important medicinal plants, with about 400 species wildly distributed in the northern hemisphere (especially in Europe, North America, Asia and South Africa) and represented in Iranian flora by 34 species (4, 5). This genus has been gaining increasing attention since the discovery of artemisinin, a promising and potent antimalarial drug which derived from the plant *A. annua* (6). Experiments suggested that artemisinin and its derivatives kill *plasmodium* protozoa by interacting with heme to produce free radicals that alkylate specific malarial proteins and damage membranes of the parasite. Moreover, artemisinin could inhibit heme bio crystallization and interact with hemozoin formation, lead to split of the malaria pigment (7, 8). Recently, the DCM extracts of *A. scoparia* and *A. spicigera* were shown to significantly inhibit the heme bio crystallization in *β*-hematin formation assay (9). In continuation of our studies on Iranian *Artemisia* species, we have now evaluated antimalarial effect of different extracts from three *Artemisia* species including *A. ciniformis*, *A. biennis* and *A. turanica. *Recently, the total extract of *A. turanica* was reported to have antimalarial effect against *Plasmodium berghei* (10). In other studies, ethanol extract of *A. turanica* has shown anticancer activity against human Caucasian hepatocyte carcinoma (HepG-2) and human Caucasian larynx carcinoma (Hep-2) cell lines (11). Moreover, methanol extract of this plant was reported to have antimicrobial activity (12). DCM extracts of *A. biennis* and *A. ciniformis* have been shown to inhibit cancer cell growth (13), likewise, different extracts of *A. ciniformis* have been reported to possess antiprolifrative effects on malignant cell lines (14, 15). It was recently reported that the ethanol extracts of these three species have inhibitory effects against *Leishmania major* parasites (16) and the hydroethanolic extract of *A. biennis* showed potent antioxidant activity in different assays (17). In the current study, the anti-malarial activity of different extracts from these three *Artemisia* species was examined by *in-vitro* *β*-hematin formation assay.

## Experimental

*Chemicals*

Hematin procine, chloroquine diphosphate, sodium dodecyle sulfate (SDS), sodium acetate, magnesium sulfate, sodium hydrogen phosphate, sodium chloride, potassium chloride, sodium hydroxide, glucose and sodium bicarbonate were purchased from Sigma-Aldrich Chemical Company, oleic acid from Fluka, dimethyl sulfoxide and hydrochloric acid from Merck and all the solvents used for extraction from Caledon and Scharlau. 

*Plant material*

The aerial parts of *A. ciniformis* Krasch. & M. Pop. Ex Poljak*, A. biennis* Willd. and *A. turanica* Krasch. were collected from Tandoreh National park, Zoshkand Sami abad, Torbat- e Jam (Razavi Khorasan province, Iran) respectively. Samples were identified by Dr Valiollah Mozaffarian (Research Institute of Forest and Rangelands, Tehran, Iran). The voucher specimens (Nos. 12569, 12570 and 12572, respectively) have been deposited in the herbarium, Department of Pharmacognosy, Faculty of Pharmacy, Mashhad University of Medical Sciences, Mashhad, Iran.

*Extract Preparation*

The plant materials were air-dried at room temperature, finely ground and extracted by maceration method (18). 100 g of each plant was extracted successively with petroleum ether (PE), DCM, EtOAC, ethanol and ethanol-water (1:1 v/v) at room temperature (Sequential maceration with ca. 3×1 L of each solvent). All the extracts were separately concentrated using a rotary evaporator at a maximum temperature of 45 °C.

*In-vitro β-hematin formation assay*

The antimalarial activity of plant extracts was evaluated by the *in-vitro* *β*-hematin formation assay described by Afshar *et al.* (9) with some modifications. Briefly, varying concentrations (0.4- 2 mg/mL in DMSO) of each extract were mixed with 3 mM of hematin, 10 mM oleic acid and 1 M HCl. The final volume was adjusted to 1 mL using sodium acetate buffer, pH 5. Chloroquine diphosphate was used as a positive control. The reaction mixtures were incubated overnight at 37 °C with constant gentle shaking. Incubation was terminated by centrifugation (14000 rpm, 10 min, at 21 °C) to collect the *β*-hematin pellets. The pellets repeatedly washed with incubation (15 min at 37 °C with regular shaking) in 2.5% (w/v) SDS in phosphate buffer saline followed by a final wash in 0.1 M sodium bicarbonate, until the supernatant was colorless. To determine the heme amount crystallized into *β*-hematin, the pellets were dissolved in 0.1 M NaOH and measured the absorbance at 400 nm (Beckman DU640 spectrophotometer). The results were recorded as % inhibition (I%) of heme crystallization compared to negative control (DMSO) using the following equation: I% = [(AN–AS)/AN]*100, where AN: absorbance of negative control; AS: absorbance of test samples.

*Statistical analyses*

All experiments were conducted in triplicate measurements and presented as the mean ± standard deviation. Data were analyzed by using SPSS, version 16.0.0 software. The IC_50_ and IC_90_values were calculated from non-linear regression analysis.

## Results and Discussion

**Figure 1:**
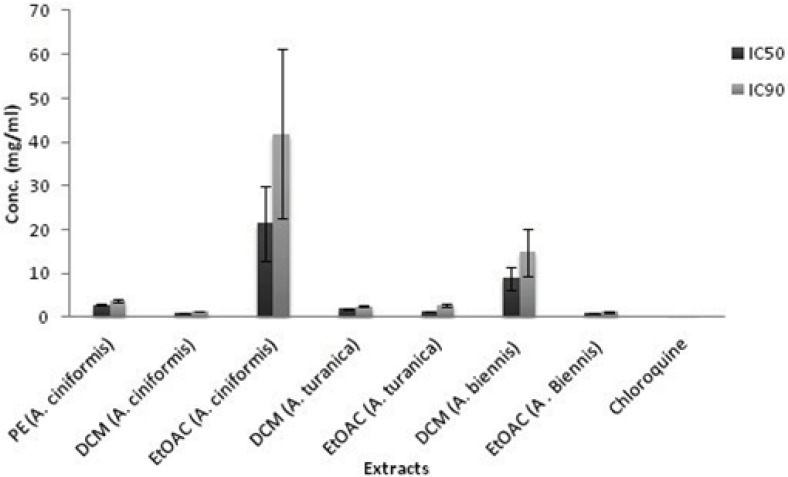
Comparison of IC_50_ and IC_90_ values (mg/mL) of active extracts of *A. ciniformis, A. turanica*, *A. biennis*, and chloroquine solution in *β*-hematin formation assay. The values were reported as Mean ± SD.

**Figure 2:**
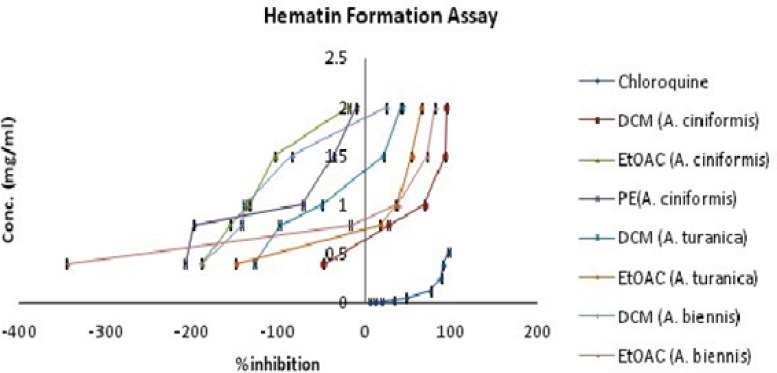
Comparison of %inhibition of heme crystallization between active extracts of *A. ciniformis, A. turanica*, *A. biennis*, and chloroquine solution in *β*-hematin formation assay. The values were reported as Mean ± SD.

During the intra-erythrocytic cycle, the malaria parasite digests the host hemoglobin within the food vacuoles of infected erythrocytes as the main source of nutrition for its development and maturation (19, 20). Massive degradation of hemoglobin is accompanied by the release of toxic free heme which affects cellular metabolism and causes parasite death (21, 22). To get rid of the excess heme, the malaria parasites have evolved a detoxification pathway which converted heme into an inert and insoluble crystal known as hemozoin or malaria pigment (23). Hemozoin bio crystallization is an essential process for the malaria parasite and is a validated target for antimalarial chemotherapy as well as drug screening programs (24). Several *in-vitro* bioassays based on differential solubility and spectral characteristics of monomeric heme and *β* -hematin (synthetic analogue of hemozoin) have been defined and exerted for searching of novel synthetic and natural antimalarial compounds (19, 24, 25). In the present investigation, the antimalarial activity was evaluated by the *in-vitro* *β*-hematin formation assay developed by Afshar *et al.* (9). The results from the antimalarial testing of fifteen extracts of *A. ciniformis, A. turanica *and* A. biennis *as well as the extraction yields are presented in Table 1. The IC_50_ and IC_90_ values for each active extract were calculated graphically by plotting concentrations against percentage of inhibition (I%) and defined as the concentration of extract causing 50% and 90% inhibition of *β*-hematin formation, respectively. As illustrated in Table 1 and Figure 1, ethanol, ethanol-water and PE extracts revealed no activities in this assay system except for PE extract of *A. ciniformis* (IC_50_ = 2.88 ± 0.26 mg/mL, IC_90_ = 3.86 ± 0.40 mg/mL), while the DCM extracts of *A. ciniformis* and *A. turanica *as well as EtOAC extracts of *A. biennis* and *A. turanica* were found to be the inhibitors of *β*-hematin formation. The most potent antimalarial activities belonged to DCM extract of *A. ciniformis* (IC_50_ = 0.92 ± 0.01 mg/mL, IC_90_ = 1.29 ± 0.02 mg/mL), followed by EtOAC extracts of *A. biennis* (IC_50_ = 1.11 ± 0.02 mg/mL, IC_90_ = 1.22 ± 0.04 mg/mL) and *A. turanica* (IC_50_ = 1.35 ± 0.08 mg/mL, IC_90_ = 2.81 ± 0.21 mg/mL).Using box and whisker plots for IC_50_ and IC_90_ values revealed the presence of an outlier that was related to EtOAC extract of *A. ciniformis.* In other words, the rest of active samples could be remained as candidates for further study and comparison. Chloroquine was tested as a reference drug with IC_50_value of 0.04 ± 0.01 mg/mL and IC_90 _value of 0.35 ± 0.01 mg/mL. It was demonstrated that compounds with potent antimalarial activity in these active extracts have medium polarity. Previous researches on natural compounds showed that terpenes, steroids (26), saponins (27), methoxylated flavonoids (28) and methylated coumarins (29) exhibited antimalarial effects in various tests. Also, according to the screening study on terpenoid content of ten Iranian *Artemisia *species carried out by Iranshahi *et al.* (30), *A. cinifomis* showed high content of sesquiterpenoid lactons while *A. biennis* and *A. turanica* have low amount of terpenes. Therefore, it seems that the potent antimalarial activity of DCM extract from *A. ciniformis* might be due to the high content of sesquiterpenoid lactones. In the case of *A. turanica* and *A. biennis*, the antimalarial activity of EtOAC extracts was superior to the corresponding DCM extracts. These results might have been derived from the high concentration of antimalarial component with higher polarity than sesquiterpenoids like methoxylated flavonoids or methylated coumarins and removing as much the lipid like compounds from these extracts. As represented in Figure 2, at lower concentrations of the potent extracts and at all concentrations (0.4-2 mg/mL) of weak extracts (PE and EtOAC extracts of *A. ciniformis*), the percent inhibition values were negative, because the observed absorbences were higher than the negative control. These data are in agreement with our previous study (9) that showed that the presence of lipids and other fatty acids in the mixture of semi-polar extracts cause synergistic effect with oleic acid in the assay. It was indicated that the IC_50_ and IC_90_ values could be decreased by entirely removing the lipids and purification of the active antimalarial compounds.

**Table 1:** The 50% and 90% inhibition concentration (mg/mL) of different extracts of *Artemisia *species in *β*-hematin formation assay

**IC**_90_ ** (mg/mL)** a	**IC**_50_ ** (mg/mL)** a	**Yields (%)**	**Extracts/Fractions**	**Plants**
3.86 ± 0.40	2.88 ± 0.26	5.31	petroleum ether	***A. ciniformis***
1.29 ± 0.02	0.92 ± 0.01	11.58	dichloromethane	
41.85 ± 19.28	21.46 ± 8.44	0.42	ethyl acetate	
-	-	3.28	ethanol	
-	-	20.72	ethanol-water	
-	-	2.74	petroleum ether	***A. turanica***
2.49 ± 0.17	1.93 ± 0.09	12.11	dichloromethane	
2.81 ± 0.21	1.35 ± 0.08	0.60	ethyl acetate	
-	-	3.85	ethanol	
-	-	18.69	ethanol-water	
-	-	5.27	petroleum ether	***A. biennis***
14.80 ± 5.50	9.02 ± 2.64	7.22	dichloromethane	
1.22 ± 0.04	1.11 ± 0.02	0.46	ethyl acetate	
-	-	1.42	ethanol	
-	-	9.94	ethanol-water	
0.35 ± 0.01	0.04 ± 0.01	-	-	**Chloroquine**

a Experiment was performed in triplicate and the results were expressed as Mean ± SD.

## Conclusion

The plant extracts in this investigation are less active antimalarials than the reference drug, chloroquine, but these extracts contain a heterogeneous mixture of various compounds and the active components might display more potent activity in their pure form. Among fifteen tested extracts, the DCM extract of *A. ciniformis* was considered more promising for further studies to isolate and identificate the active antimalarial principles. 
